# Short Copy Number Variations Potentially Associated with Tonic Immobility Responses in Newly Hatched Chicks

**DOI:** 10.1371/journal.pone.0080205

**Published:** 2013-11-25

**Authors:** Hideaki Abe, Kenji Nagao, Miho Inoue-Murayama

**Affiliations:** 1 Wildlife Research Center, Kyoto University, Kyoto, Japan; 2 Department of Anatomy, University of Otago, Dunedin, New Zealand; 3 Animal Husbandry Research Division, Aichi Agricultural Research Center, Aichi, Japan; Auburn University, United States of America

## Abstract

**Introduction:**

Tonic immobility (TI) is fear-induced freezing that animals may undergo when confronted by a threat. It is principally observed in prey species as defence mechanisms. In our preliminary research, we detected large inter-individual variations in the frequency and duration of freezing behavior among newly hatched domestic chicks (*Gallus gallus*). In this study we aim to identify the copy number variations (CNVs) in the genome of chicks as genetic candidates that underlie the behavioral plasticity to fearful stimuli.

**Methods:**

A total of 110 domestic chicks were used for an association study between TI responses and copy number polymorphisms. Array comparative genomic hybridization (aCGH) was conducted between chicks with high and low TI scores using an Agilent 4×180 custom microarray. We specifically focused on 3 genomic regions (>60 Mb) of chromosome 1 where previous quantitative trait loci (QTL) analysis showed significant F-values for fearful responses.

**Results:**

ACGH successfully detected short CNVs within the regions overlapping 3 QTL peaks. Eleven of these identified loci were validated by real-time quantitative polymerase chain reaction (qPCR) as copy number polymorphisms. Although there wkas no significant *p* value in the correlation analysis between TI scores and the relative copy number within each breed, several CNV loci showed significant differences in the relative copy number between 2 breeds of chicken (White Leghorn and Nagoya) which had different quantitative characteristics of fear-induced responses.

**Conclusion:**

Our data shows the potential CNVs that may be responsible for innate fear response in domestic chicks.

## Introduction

Tonic immobility (TI) is an unlearned defensive behavior characterized by temporal paralysis [1], and is widely used to measure the extent of fear responses in chickens [2], [3] and other animals [4]–[6]. An individual with fewer induction attempts and longer TI responses is generally considered to be more fearful than those with many induction attempts and shorter TI responses [7]. Indeed, previous studies have demonstrated that the Red Junglefowl (RJF) and domesticated White Leghorn (WL) can be discriminated by different quantitative distributions of TI indices [8], [9]. Our previous study also detected significant differences in the TI responses between WL and Nagoya breeds (NG) in newly hatched chicks (days 1–2 after hatching) [10]. These significant levels of interbreed heterogeneity may be attributed to the artificial selection of response insensitivity to human handling during the process of chicken domestication.

Considerable efforts have been taken to understand the molecular basis of anxiety and fear-based responses based on the hypothesis that genetic linkage or pleiotropic gene effects could explain different reactions to fearful stimuli. In chickens, there are 2 major quantitative trait loci (QTL) for individual growth on chromosome 1 (*Growth1* and *Growth2*) [11], and surprisingly, *Growth1* QTL contains several genes which, together, affect personality [8]. Moreover, an important finding has been made regarding genetic links between fear responses and major growth QTLs in an RJF × WL intercross [7]. These findings raise the possibility that the growth QTL may contain genes or genetic regions that influence the extent of fear-related behavior in chickens with far-reaching effects at the molecular and cellular levels.

Another effective and reliable approach for identifying genes or genomic regions responsible for normal behaviors is to perform genome-wide searches for copy number gains and losses. Copy number variation (CNV) is defined as genomic duplications or deletions in relatively long elements (1 kb to several Mb in size). With increasing resolution in the detection of smaller CNVs, this definition has expanded to include short structural variants less than 1 kb, known as short CNVs (sCNVs) [12]. In humans, CNVs have been linked to various behaviors including brain-related disorders [13]. In non-human vertebrates, including chickens, a growing number of studies have focused on the associations between CNVs and observed phenotypic heterogeneities [14], and thus CNV has been recently recognized as an important source of genetic variability that may affect phenotypes because of the rearrangement of the genes or regulatory elements.

The main goal of this study was to identify novel sCNVs between chicks with high and low TI scores by using an array comparative genomic hybridization (aCGH) approach. We targeted 3 different QTL in chromosome 1, for which significant *F* values had been detected for TI responses in chickens. Our approach provides an efficient way to narrow the number of plausible factors that account for differences in fear-induced behaviors by focusing on the regions containing interesting QTL.

## Materials and Methods

### Animals Used in this Study

We used 3 breeds/strains of chicken with different selection histories (NG5 [*n = *32], NG7 [*n = *39], and WL [*n = *39]). NG was chosen as the target chicken breed in this study because of the following reason: NG chicks occasionally panic and are hurt when they are frightened by sounds or small stimuli. It is especially important for future management of economically significant breeds to uncover their genetic basis of fear-related behaviors.

The NG breed was originated from a cross between a local chicken from the City of Nagoya and the Chinese Buff Cochin in the early 1880 s. In 1905, this breed was recognized as the first practical breed for poultry farming in Japan, and the NG was formally established in 1919 [15]. Of the various strains, NG5 and NG7 have distinct histories of selection either as a layer-type strain (NG5) or as a meat-type strain (NG7). The details of husbandry of chicks have been described elsewhere [10].

### Tonic Immobility Test

A TI test was conducted using male chicks on days 1 and 2 after hatching. We measured TI responses 6 times for each chick (3 times on each of the days 1 and 2), regardless of the success rate of TI induction. We employed the same method used for assessing TI scores in adult chickens [1]; each chick was placed on its back in a V-shaped cradle and was kept there with light pressure on its breast for 5 s. After removing the pressure, chicks were not considered to be in TI status if the bird jumped up or righted itself within 5 s. We carried out the procedure 3 times in succession on each individual. The operator recorded the number of induction attempts required to induce a chick into the TI status as well as the time until righting in each successful TI induction (hereafter expressed as TI_ind_ and TI_dur_, respectively). If a bird did not enter TI status for all 6 attempts, TI_ind_ was assigned a score of 7. When 10 min had passed since the bird entered TI, the chick was forced to stand up, and TI_dur_ was scored as 600 s. A fixed video camera was also used to ensure that environmental factors such as unexpected noise and changes in light intensity had no influence on chicks’ fear-relevant behaviors.

### Microarray Design

Blood samples were collected from the 3 chicken breeds/strains of chickens and stored at −20°C until DNA extraction. We isolated genomic DNA from 110 chicks by using either the PUREGENE® DNA Purification Kit (Gentra Systems, Minneapolis, USA) or DNeasy® Blood and Tissue Kit (Qiagen, Tokyo, Japan). DNA concentration of each extract was measured using a NanoDrop spectrophotometer (NanoDrop Technologies, Wilmington, DE). Four individuals with the highest average TI_dur_ were chosen from NG (IDs: NG933 [TI_ind_ = 1; average TI_dur_ = 248.8 s], NG4692 [TI_ind_ = 1; average TI_dur_ = 172.3 s], and NG3557 [TI_ind_ = 1; average TI_dur_ = 255.0 s]) and WL (WL3597 [TI_ind_ = 1; average TI_dur_ = 199.3 s]) strains as samples compared in aCGH analysis. One NG chick (NG999 [TI_ind_ = 7; TI_dur_ = 0]) that had not been induced the TI status in all 6 attempts was chosen as the reference sample. We used a chicken CGH Microarray 4×180K (Agilent Technologies, Tokyo, Japan), containing 180,000 custom probes of 60-mer, because Agilent’s 60-mer offers the highest sensitivity and reproducibility among the currently available commercial platforms [16]–[18]. We designed these probes by using the eArray software (Available: https://earray.chem.agilent.com/earray/Accessed 2011 May 19). Note that these probes covered a total of 60 Mb in exonic, intronic, and intergenic regions of chromosome 1, where significant *F* values were detected by previous QTL analysis for fear-related behaviors [7]. Information on QTL for TI attempts (trait ID: 2123) and duration (2124) in the chicken genome was obtained from the QTL database (Available: http://www.genome.iastate.edu/cgi-bin/QTLdb/GG/index. Accessed 2011 Oct 11). The mean probe spacing was 1,029 bp, and the median probe spacing was 264 bp. Our strategy was somewhat analogous to that employed by a previous study [19], which targeted for restricted chromosomal regions in the porcine genome. There were several reasons for targeting sCNVs as a candidate for TI response variability. Although no clear pattern for CNV effect versus CNV-gene distance has been observed, smaller variants less than 1 kb have been found to be more likely to regulate gene transcription than larger variants [12]. Moreover, a recent study suggested that sCNVs tend to originate from the presence of a variable number of tandem repeats, which could provide a source of genetic variability for modifying normal and abnormal human behaviors [20]. All hybridizations were performed using 2 dyes for labeling reference (Cy3) and sample DNA (Cy5). The hybridization and initial data analysis were performed by MACS® Molecular genomic service (Miltenyi Biotec GmbH, Bergisch Gladbach, Germany).

### Detection of Copy Number Variations

Statistical analysis for CNV detection was performed using Agilent GENOMIC WORKBENCH Standard Edition 6.5 software (Agilent Technologies). The minimum number of probes present in an aberrant region was 4, and aberrant segments were identified for a CNV locus when the average log_2_ ratio was greater than |±0.4|. In addition, a less stringent filter was used to infer aberrant regions under the condition that the minimum number of probes present in an aberrant region was 2. In both cases, the statistical analysis of aberrant regions was based on the aberrant detection algorithm ADM-2. The full data set and designs from the oligo aCGH experiments have been submitted to the GEO database [21] under the accession ID GSE38434.

### Copy Number Validation by Quantitative Polymerase Chain Reaction

To validate representative aCGH results, quantitative polymerase chain reaction (qPCR) was performed using the chicken β-actin gene (*ACTB*) as a reference for normalization of the real-time PCR experiments. On the basis of the aCGH aberration data, 52 sets of PCR primers for candidate sites were designed using Primer3 software [22]. Each set of primer pair was expected to yield PCR products, ranging from 150 to 200 bp based on the sequence in the chicken genome assembly build 3.1. The primer sequences are listed in Table S1. Prior to real-time PCR, each product was electrophoresed on a 2.0% agarose gel in order to verify the expected product size. Amplification curve and Ct values were generated with the Thermal Cycler Dice® Real Time System (Takara Bio, Shiga, Japan). Each qPCR reaction (15 µL) contained 10 ng of genomic DNA, 2.0 µL of 5 µM forward and reverse primers, 0.3 µL of ROX reference dye, and 7.5 µL of the 2× SYBR® Premix Ex Taq™ II (Takara Bio). The PCR cycle consisted of initial denaturation at 95°C for 30 s, followed by 40 cycles at 95°C for 5 s and 60°C for 31 s. Each sample was run 3 times to obtain accurate qPCR results. For relative amount quantification, Ct value differences were used to quantify the relationship between relative copy number and β-actin. This was calculated as follows: relative copy number = log2^ΔCt^, where ΔCt = Ct_β-actin_ – Ct_target_. The Ct values obtained were in agreement within and between runs.

### Statistical Analysis

Since almost all test data showed non-normal frequency distributions that could not be transformed to meet the requirements of parametric statistical analysis, the Kruskal-Wallis test was carried out to examine the difference in TI_ind_ and TI_dur_ scores between breeds/strains. In each sCNV locus, we compared TI scores with the distribution of relative copy number, calculated as log2^ΔCt^. To compare relative copy number between chicks with high and low TI scores, each chicken cohort was classified into high (TI_ind_ = 1; TI_dur_ ≥60 s) and low (TI_ind_ = 2∼7; TI_dur_ <60 s) groups. Biased relationships between copy number and each TI score were examined using *F*-statistics under the null hypothesis of no association between copy number on the target locus and TI responses.

### Ethical Note

All aspects of the study were performed according to the guidelines established by the Ministry of Education, Culture, Sports, Science and Technology in Japan (Notice No. 71). This study fulfilled ethical guidelines of the International Society of Applied Ethology [23]. The protocol was approved by the Committee on the Ethics of Animal Experiments of the Wildlife Research Center of Kyoto University (Permit No. WRC-2012-EC001).

## Results

**Figure 1:**
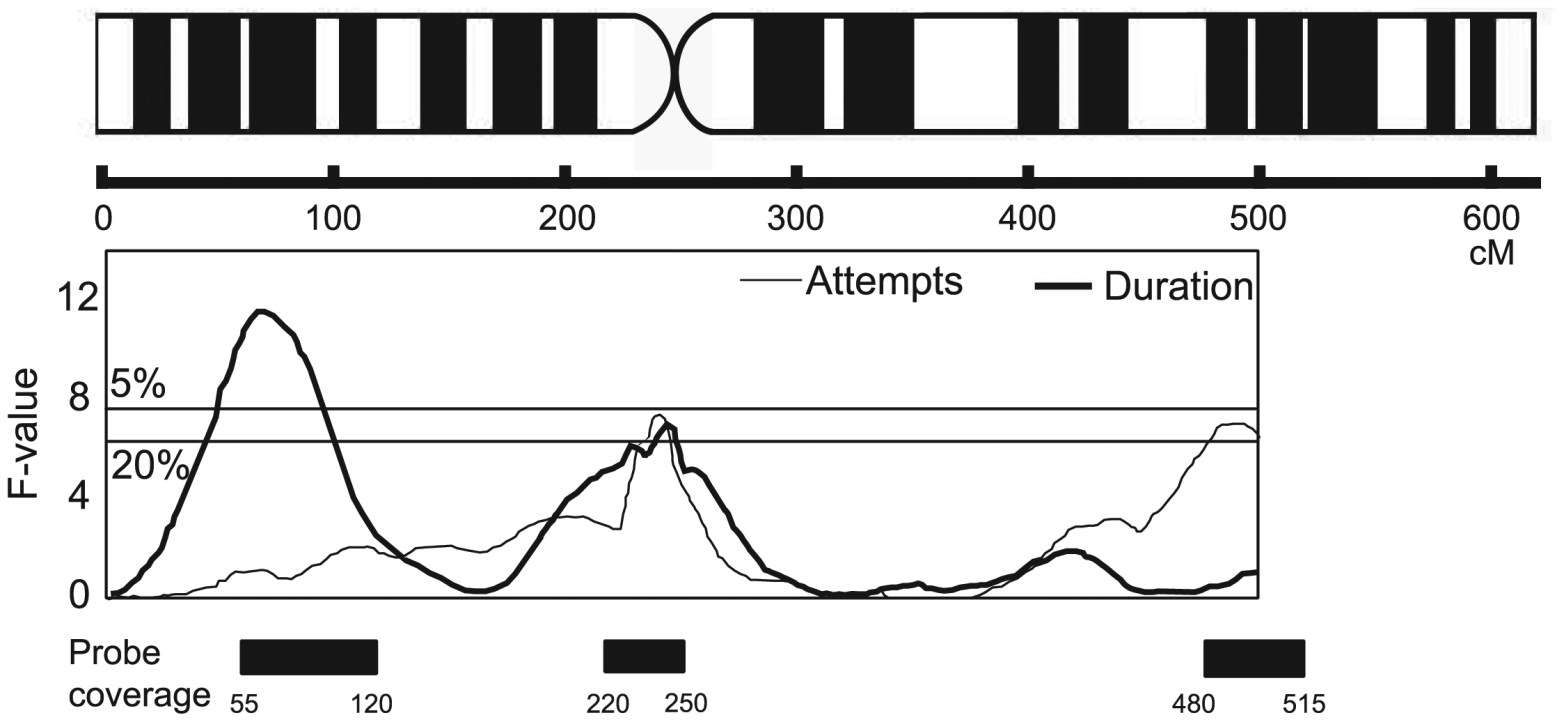
Probe coverage on chicken chromosome 1 for array comparative genomic hybridization. Probes are designed in 3 regions (>60 Mb) where significant *F*-values have been identified by previous quantitative trait loci analysis. Genome-wide *F*-values for tonic immobility duration (thick line) and induction attempts (thin line) are quoted from [7].

A total of 180,000 unique probes was designed in chicken chromosome 1 targeting 3 identical QTL for fear traits (Figure 1). Table 1 shows the main results from the TI experiment in each cohort of newly hatched chicks. WL chicks were easily induced into TI status according to the number of successful TI inductions to total attempts and TI_ind_ as compared with those of NG (*F*_1,107_ = 16.18; *p*<0.001), whereas TI_dur_ in WL was significantly shorter than that of NG (*F*_1,107_ = 4.56; *p*<0.05). Data from NG5 and NG7 were combined for statistical analysis, since we did not find any difference in their TI scores (*p*>0.05).

**Table 1:** The induction and duration of TI response in each chicken breed/strain.

Strains	*n*	Induction/attempts	Induction	TI duration		
NG5	32	51/192 (0.27)	4.4	99.4	±	15.8
NG7	39	45/234 (0.19)	4.6	114.2	±	22.5
WL	39	82/234 (0.35)	2.7	74.8	±	10.2

**Note**: Standard error of the mean (SEM) are shown with the time until righting (sec).

A large number of aberrant loci were detected in chicken chromosome1 based on the aCGH analysis between chicks characterized with high and low TI scores. The total number of aberrant segments identified in the 4 comparisons was 202 (average 50.5) in a stringent setting (4 probes) and 477 (average 119.3) in a less stringent setting (2 probes). Of these segments, 288 showed loss variation, and the remaining 391 segments showed gain variation. The duplicated segments (gain) occupied 57.6% in total length aberration. The average length of gain or loss segments was estimated at 3,552 bp (4 probes) and 1,833 bp (2 probes). The 477 CNVs found under the less stringent criteria encompassed 874.4 kb, which accounted for 1.46% of the total target region (60 Mb) in this study. This ratio was similar to the value suggested by whole genome analysis, indicating that CNVs occupied 1.34% of the entire chicken genome [24]. CNVs were not equally distributed throughout the target regions; the distal QTL peak (480–515 cM) of chromosome 1 contained a greater number of sCNVs than the other 2 QTL regions (Fisher’s exact test; *p*<0.01). Since a previous study also detected a large number of CNVs in this region [24], this chromosomal region can be regarded as a “CNV hotspot” in which mutations leading to copy number differences between individuals occur more frequently than expected. We further screened these aberrant segments under the following two conditions: the segments were commonly detected in all genomic comparisons with log_2_ ratio |±0.4|, and the segments were detected in at least 2 comparisons with log_2_ ratio |±1.2|. From the whole data set (4 & 2 probes), we extracted 52 loci that satisfied either of these conditions.

Real-time qPCR was performed to validate the aCGH data for 52 candidate loci. For preliminary screening procedure of qPCR validation, 12 DNA samples from NG and WL strains, including samples used for aCGH analysis, were employed as templates. The qPCR analysis displayed different patterns of quantitative variations that could be classified into 5 categories: (1) the same level of ΔCt values was detected in all samples except for the reference sample, whose PCR product was completely absent (described as “deletion” in Table 2); (2) an apparent variation of the ΔCt values was observed in 12 samples including the reference sample (described as “CNV” in Table 2) (3) almost the same level of ΔCt values was observed in all samples including a reference sample, i.e., monomorphic loci; (4) no PCR product was detected in several samples including or excluding the reference sample; and (5) failed PCR amplification in all specimens, probably due to primer mismatch. We found quantitative variations in the following percentage: (1) 11.5%, (2) 9.6%, (3) 63.5%, (4) 3.8%, and (5) 11.5%. We identified 11 loci, belonging to either category (1) or (2), as candidate sCNVs, and sCNVs in the other category were briefly summarized in Table S2.

**Table 2:** Candidate short Copy Number Variations identified by array Comparative Genomic Hybridization and subsequent qPCR validation.

locus ID	probes	Start	Stop	bp	Gene	qPCR
TIC_03	2	25773437	25773746	309	Non-coding	CNV
TIC_04	2	28079752	28080201	449	Non-coding	CNV
TIC_05	2	28432127	28432740	613	Non-coding	deletion
TIC_15	2,4	87064880	87065977	1097	NME7	deletion
TIC_16	2	170375957	170376395	438	KIAA0564	CNV
TIC_18	2,4	176058363	176059210	847	Non-coding	CNV
TIC_42	2	177404311	177404572	261	NBEA	CNV
TIC_44	2	180954362	180954612	250	CDK8	CNV
TIC_19	2	181380041	181380759	718	NUPL1	CNV
TIC_20	2,4	186702464	186703251	787	Non-coding	CNV
TIC_21	2	188457807	188458430	623	Non-coding	CNV

**Note**: Only loci displaying quantitative difference in qPCR validation are shown here.

**Figure 2:**
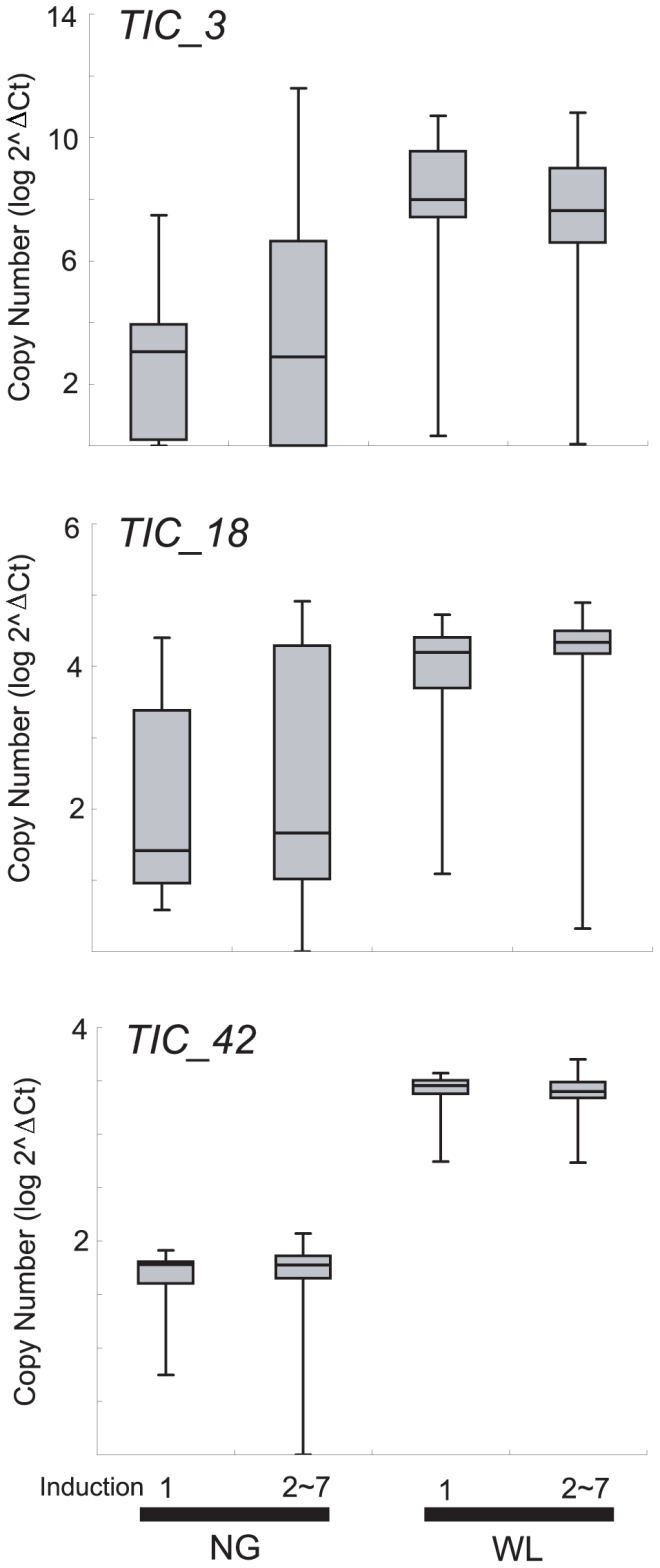
Comparison of relative copy number between chicken breeds with different sensitivity to fear. Relative copy number is calculated as log2^ΔCt^, where ΔCt = Ct_β-actin_ – Ct_target_. NG and WL indicate Nagoya and White Leghorn, respectively. The number of samples in each group was; *n* = 13 (NG; TI induction 1), *n* = 58 (NG; TI induction 2∼7), *n* = 13 (WL; TI induction 1), and *n* = 26 (WL; TI induction 2∼7).

For the 11 candidate loci, we examined the association between sCNVs and TI_ind_ or TI_dur_ scores in 110 chicks. With regard to both TI indices, we detected significant differences in the copy number distributions between NG and WL in *TIC_3* (*F*_1,107_ = 4.10; *p*<0.05), *TIC_18* (*F*_1,107_ = 19.05; *p*<0.001), and *TIC_42* (*F*_1,107_ = 236.59; *p*<0.001; Figure 2). However, there was no difference in the relative copy number for all target CNVs between chicks with high and low TI scores (TI_ind_ & TI_dur_) in each NG and WL. Scatter plots of correlation analysis between TI scores and the relative copy number in each locus were shown in the supporting information (Figures S1 and S2).

## Discussion

Until now, most studies on chicken domestication have focused on the genetic and behavioral heterogeneities between RJF and WL in order to highlight the alternative histories of domestication. Additionally, our preliminary study [10] detected large differences in the quantitative traits of TI responses between WL and NG populations. WL chicks were prone to be induced into the TI status with fewer attempts (low TI_ind_), whereas NG had longer TI_dur_ in each successful TI induction. These empirical data strongly support the previous hypothesis that the TI behavior in chickens has a genetic basis with breed- or strain-specific behavioral characteristics [8], [25], [26].

However, we did not detect a difference in the relative copy number between high and low TI groups within each chicken breed. There are several reasons for this, one of which is that the number of chicks used in this study may be insufficient to detect an association between sCNVs and variations in the chicks’ sensitivity to fearful stimuli. Considering that a TI response is not simple but has a complicated contextual framework involving cognitive and neural processes, there may be multiple genetic determinants associated with the quantitative traits of TI responses. Therefore, to detect an effect of CNVs on TI, sample sizes would need to be increased more than 20-fold over the current study design, and other genetic and epigenetic factors such as single nucleotide polymorphisms (SNPs) and methylation patterns should be included as possible candidates for affecting fear-induced behaviors in future research. Another explanation for the lack of significant results in this study is the inconsistency in chicken breeds between the reference genomic DNA sequence derived from NG and the genome shotgun sequence of RJF [27] used for primer design in qPCR validation. Based on the patterns of qPCR amplification, more than half (63.5%) of candidate CNV loci showed monomorphism in amplification plots. This is probably due to considerable sequence diversity between NG/WL and RJF, which might lead to an apparent loss of copy number polymorphisms in the validation phase. According to archaeological findings [28], the divergence time of domestic chickens from RJF is estimated to be nearly 8,000 years. Comparison of DNA sequences from 30 introns at 25 nuclear loci revealed that the extent of nucleotide divergence after the split of RJF from their chicken ancestor is as small as 0.01% [29]. However, a more recent study indicated that NG lines were genetically distinct from commercial gene pools, thus making it a unique genetic resource [30]. A way to avoid this complication would be to change the strategy by including target chicken breeds in TI measurements. Finally, we cannot exclude the possibility that innate and learned fear responses are modulated differently by independent neural networks and mechanisms. It should be emphasized that in previous QTL analysis, TI tests were conducted when chickens were 29–30 weeks of age [7], whereas newly hatched chicks were used here for TI testing to preclude secondary social and environmental effects on TI responses. Therefore, if TI scores would fluctuate during the growing stages of chicks and juveniles, as has been suggested by previous studies [31], [32], these conflicts may have some impact on the outcome of genome-wide association studies (GWAS) between genetic variants and quantitative TI scores. We therefore consider our aCGH approach for analyzing inter-individual variations in freezing behavior, a useful preliminary analysis capable of generating further hypotheses for future evaluation.

Among the candidate sCNVs, which were excluded by aCGH screening and subsequent qPCR, we detected significant differences in the relative copy number between NG and WL in *TIC_3*, *TIC_18*, and *TIC_42*. The *TIC_3* locus is embedded within *Growth1* QTL, for which the highest *p* value was obtained in a QTL study for TI traits [7]. The fact that this locus was found in a relatively large non-coding region may imply that it affects gene expression through far-reaching cis- or trans-acting mechanisms. The *TIC_18* locus is located upstream of the *TRPC4* gene (*ENSGALG00000017044*), which plays a role in multiple processes, including neurotransmitter release and exocytosis [33]. A recent study suggested that TRPC4 play a pivotal role in regulating dopamine release, which may modulate emotional and cognitive responses in rats [34]. Thus, it is worthwhile investigating the expression of orthologous genes in the chicken brain and further examining the correlation between levels of expression and innate fear responsiveness. The other candidate sCNV (*TIC_42*) was found in the *NBEA* gene (*ENSGALG00000017062*), which has been identified as a putative regulator of membrane protein trafficking associated with the *trans*-Golgi network. [35]. NBEA-deficient mice died immediately after birth apparently from respiratory paralysis [36]. NBEA plays a complex role in the development and functioning of synapses and is believed to play a role in autism spectrum disorders by evoking an excitatory-inhibitory imbalance in synaptic activity [37]. Further information on differential gene expression of these candidate genes in the brain between various chicken selection lines, will provide opportunities to examine their role in shaping behavioral differences.

In conclusion, we identified 11 sCNVs that potentially account for the robust differences observed in TI responses among chicken breeds. None of the genes identified in this study have been directly implicated in TI responses; however, studies on several of these genes, such as *TRPC4* and *NBEA*, have reported brain functions that might be involved in abnormal behaviors in humans and rodents. Future experiments involving gene expression assays and mutagenic approaches are needed to determine whether any of the novel candidate genes identified here play a role in modulating innate fear responses.

## Supporting Information

Figure S1 **Correlation analysis between the induction score of Tonic immobility (TI_ind_; x-axis) and relative copy number (ΔCt; y-axis).**
(PDF)

Figure S2 **Correlation analysis between the duration score of Tonic immobility (TI_dur_; x-axis) and relative copy number (ΔCt; y-axis).**
(PDF)

Table S1 **List of primers that show copy number polymorphisms in qPCR.**
(PDF)

Table S2 **Another sets of candidate short Copy Number Variations examined in this study.**
(PDF)
