# Supplementary figures and images for: Short Copy Number Variations Potentially Associated with Tonic Immobility Responses in Newly Hatched Chicks
Source: PLoS One. 2013 Nov 25;8(11):e80205. doi: 10.1371/journal.pone.0080205 (PMC3839970; doi:10.1371/journal.pone.0080205)

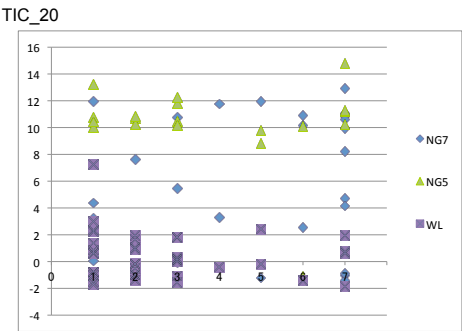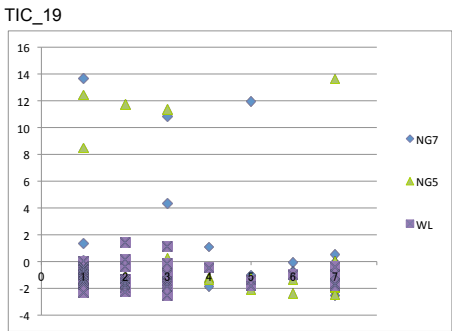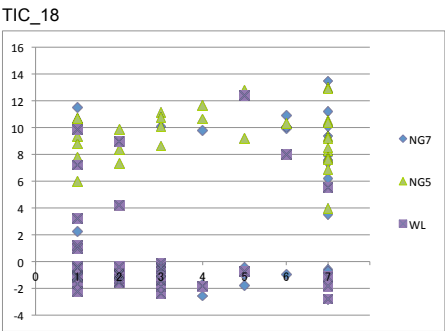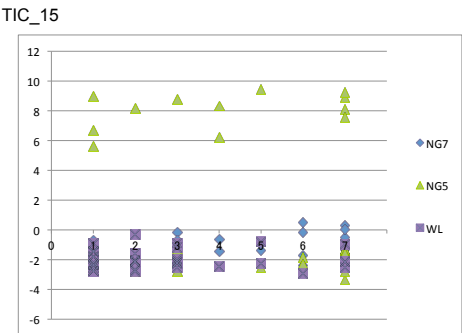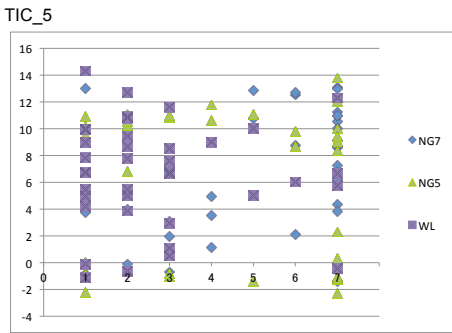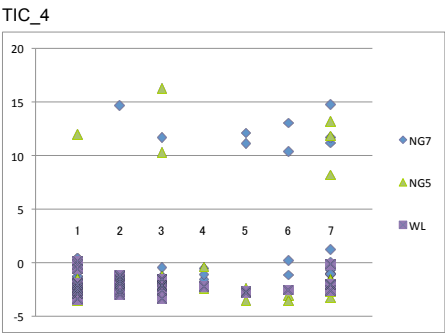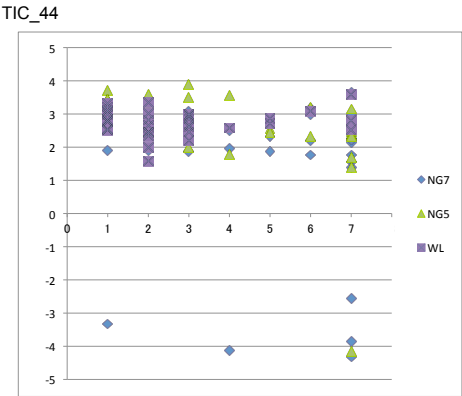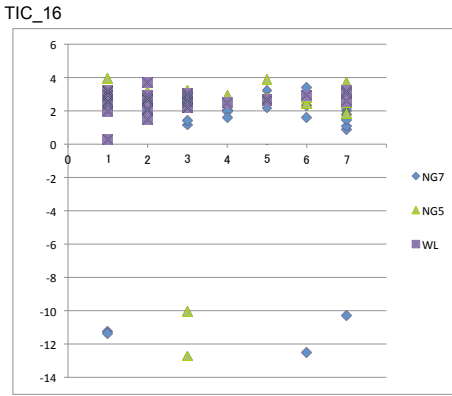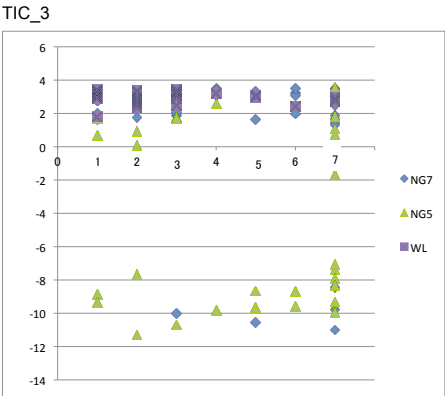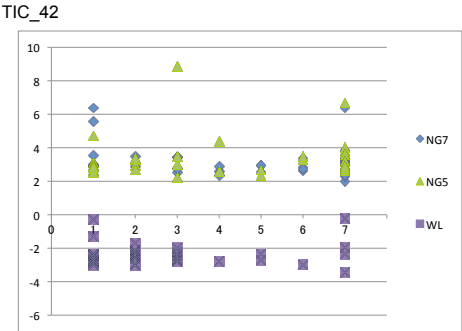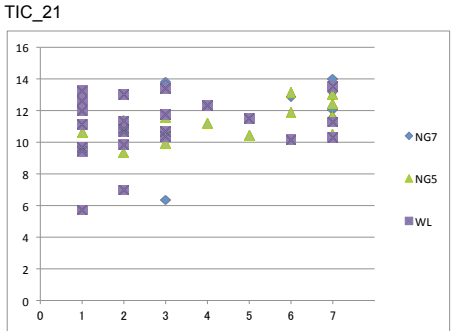

Figure S1

Supplement: Figure S1 — Correlation analysis between the induction score of Tonic immobility (TIind; x-axis) and relative copy number (ΔCt; y-axis). (PDF) [file pone.0080205.s001.pdf]

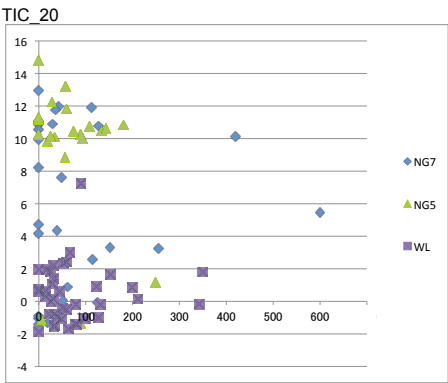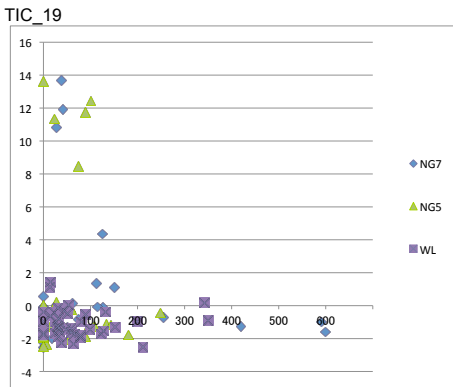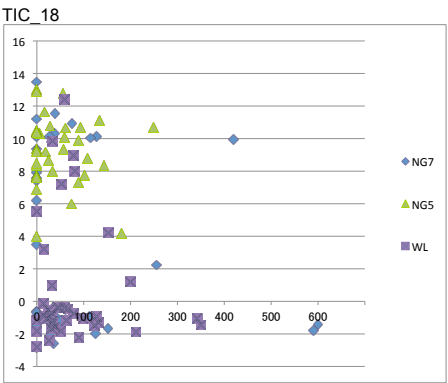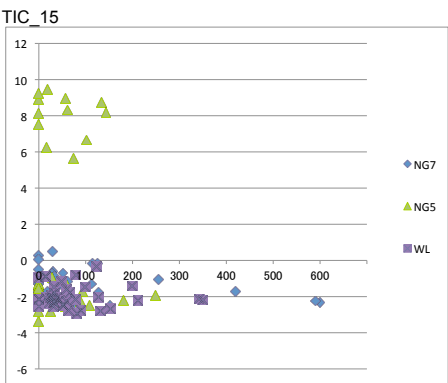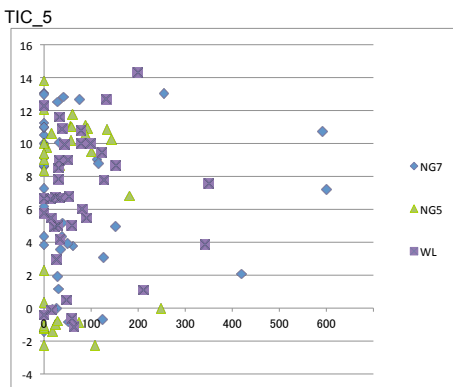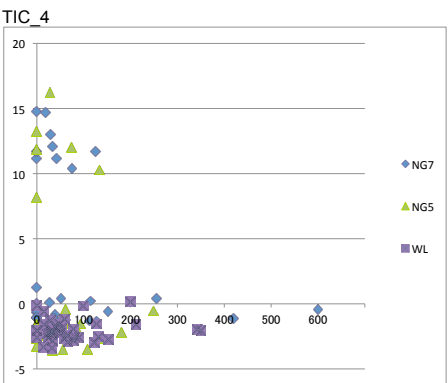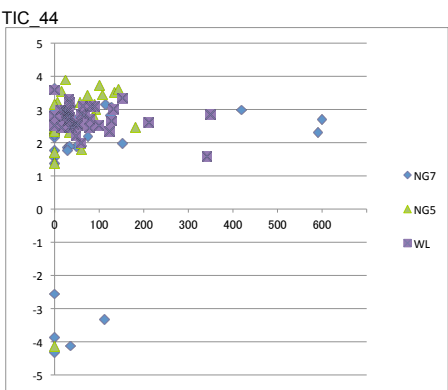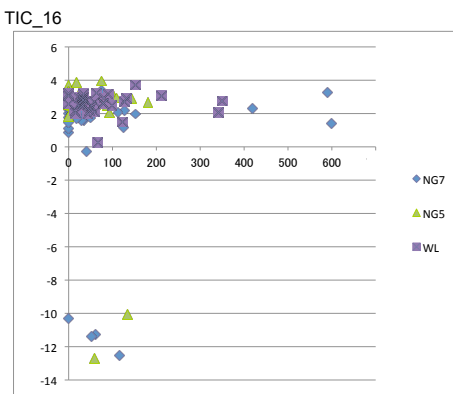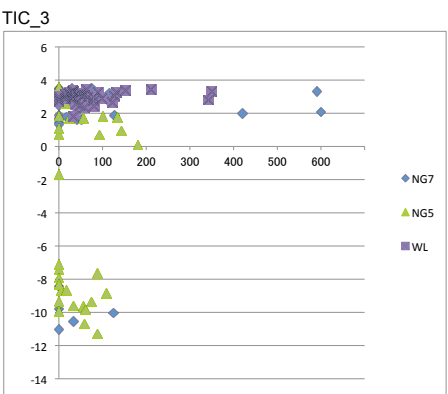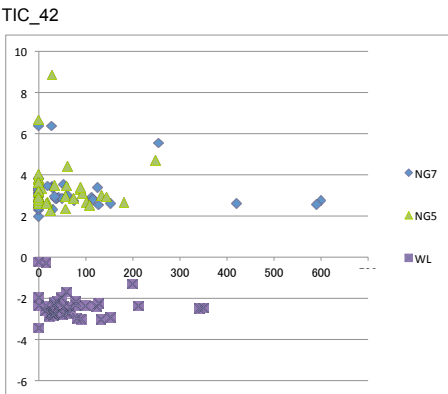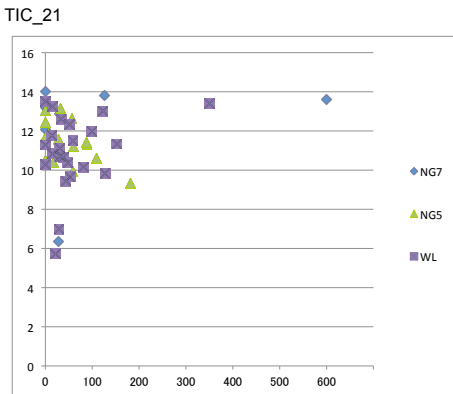

Figure S2

Supplement: Figure S2 — Correlation analysis between the duration score of Tonic immobility (TIdur; x-axis) and relative copy number (ΔCt; y-axis). (PDF) [file pone.0080205.s002.pdf]
