# Supplementary material for: Short Copy Number Variations Potentially Associated with Tonic Immobility Responses in Newly Hatched Chicks
Source: PLoS One. 2013 Nov 25;8(11):e80205. doi: 10.1371/journal.pone.0080205 (PMC3839970; doi:10.1371/journal.pone.0080205)
Supplement: Table S1 — List of primers that show copy number polymorphisms in qPCR. (PDF) [file pone.0080205.s003.pdf]

Supplementary Table1

List of primers used for quantitative PCR validation

Locus	Forward (5'–3')	Reverse (5'–3')
TIC_3	CCAAACCAGATTGGAAGGAA	GGAGAGTGTTGGTCTCACTCCT
TIC_4	TTCCACGTTTGCTAGTAACGAC	CCAAGCTGAGGTGGCTCTAC
TIC_5	GAATGCCATGGAAATCCAAA	TCGTGGAGAGGGTTTTGTTC
TIC_15	AAACCAAGCCAGAAAGCAGA	GCTCTTTGCTTGGGAAGATG
TIC_16	AGCAGTGACACTTGCCTTGA	TCTACTCCTGTGATGGGTTGG
TIC_18	GCCCAAATGCCTTTAGATGA	ACTTGGGAGAAGGGAGCATT
TIC_19	CTCTTCTCCCACCCACTTCA	TGAAACACCAGCAACTGAGC
TIC_20	AAGCAAGCCCAGAAAAGTCA	GGGTGGCACACATGATGTTA
TIC_21	TCTTTGCAACATAGCCCACA	TCACCAGTGGGAGACAGAAA
TIC_42	AGCCTGGTGAACAGAGGTGT	AGGGGGACTCACAGTTGATG
TIC_44	AGCAAGAGCAGGGAAGTGAT	ACTTGTGCTCCAGACCTTGC
ACTB*	TGCGTGACATCAAGGAGAAG	CCAAGAAAGATGGCTGGAAG

* ACTB (β-actin) is chosen as a reference locus to standardize Ct value of each sample
