# Supplementary material for: Short Copy Number Variations Potentially Associated with Tonic Immobility Responses in Newly Hatched Chicks
Source: PLoS One. 2013 Nov 25;8(11):e80205. doi: 10.1371/journal.pone.0080205 (PMC3839970; doi:10.1371/journal.pone.0080205)
Supplement: Table S2 — Another sets of candidate short Copy Number Variations examined in this study. (PDF) [file pone.0080205.s004.pdf]

Table S2 Another sets of candidate short Copy Number Variations examined in this study

loci*	#Probe	ProbeName	Start	Stop	bp	Description
TIC_01	2,4	A_94_P10089386-90	22872846	22873829	983	Unknown
TIC_02	2,4	A_94_P10096868-73	24753436	24754586	1150	Unknown
TIC_06	2	A_94_P10130437	32904537	32904596	59	Unknown
TIC_07	2,4	A_94_P10135829-884	34305780	34320308	14528	Unknown
TIC_08	2,4	A_94_P10160758	40358597	40358656	59	Unknown
TIC_09	2,4	A_94_P10311218-46	79525472	79541628	16156	Unknown
TIC_10	2,4	A_94_P10313882	80234725	80234784	59	inhibitor of growth family, member 4 (ING4), mRNA.
TIC_11	2,4	A_94_P10316803-08	81006736	81008356	1620	Unknown
TIC_12	2	A_94_P10319555-57	81811178	81812063	885	Unknown
TIC_13	2	A_94_P10326890-02	83636405	83636963	558	Unknown
TIC_14	2,4	A_94_P10332758-60	85062055	85062470	415	mRNA for chLAMP, g9-isoform.
TIC_17	2,4	A_94_P10659203	170385908	170385967	59	Unknown
TIC_22	2	A_94_P10736575-77	190339899	190340664	765	finished cDNA, clone ChEST767d1.
TIC_23	2	A_94_P10076182-83	19536495	19536737	242	Unknown
TIC_24	2	A_94_P10078838-40	20226034	20226523	489	Unknown
TIC_25	2,4	A_94_P10083862-65	21512802	21513741	939	Unknown
TIC_26	2	A_94_P10089202-04	22828030	22828536	506	Unknown
TIC_27	2,4	A_94_P10098208-215	25086631	25088097	1466	Unknown
TIC_28	2	A_94_P10101898-99	26014926	26015212	286	Unknown
TIC_29	2	A_94_P10102375-76	26141518	26141779	261	Unknown
TIC_30	2	A_94_P10108919-20	27676955	27677474	519	Unknown
TIC_31	2	A_94_P10113167-68	28683397	28683629	232	Unknown
TIC_32	2	A_94_P10117902-03	29874452	29874793	341	Unknown
TIC_33	2	A_94_P10135031-32	34072581	34072939	358	Unknown
TIC_34	2	A_94_P10139876-78	35330595	35331030	435	SLIT-ROBO Rho GTPase activating protein 1 (SRGAP1), mRNA.
TIC_35	2	A_94_P10152724-25	38388598	38388927	329	tryptophan hydroxylase 2 (TPH2), mRNA.
TIC_36	2	A_94_P10169173-74	42380167	42380480	313	finished cDNA, clone ChEST384n3.
TIC_37	2	A_94_P10309480-81	79031407	79031637	230	Unknown
TIC_38	2	A_94_P10324067-69	82937966	82938453	487	Unknown
TIC_39	2	A_94_P10677470-71	175076255	175078030	1775	Unknown
TIC_40	2,4	A_94_P10681160-64	176020653	176021692	1039	Unknown
TIC_41	2	A_94_P10683280-81	176583535	176583769	234	family with sequence similarity 48, member A (FAM48A), mRNA.
TIC_43	2	A_94_P10699818-20	180745478	180746026	548	Unknown
TIC_45	4	A_94_P10702860-64	181493755	181495047	1292	Unknown
TIC_46	2	A_94_P10719022-23	185652188	185652528	340	glutamate receptor, ionotrophic, AMPA 4 (GRIA4), transcript variant 1, mRNA.
TIC_47	4	A_94_P10719880-85	185883061	185884113	1052	Unknown
TIC_48	4	A_94_P10725016-28	187206942	187209729	2787	Unknown
TIC_49	2	A_94_P10726129-30	187502866	187503172	306	Unknown
TIC_50	2	A_94_P10727904-06	187990621	187991140	519	contactin 5 (CNTN5), mRNA.
TIC_51	2	A_94_P10730749-52	188810333	188811027	694	Unknown
TIC_52	2,4	A_94_P10742305-09	191846266	191847146	880	Unknown

Note: These loci did not show any quantative difference in validation PCR (see text for details).
